# Supplementary figures and images for: High-Throughput Screening Identifies Small-Molecule Inhibitors of the Tau-LRP1 Interaction
Source: bioRxiv. 2026 Jun 25:2026.06.24.733881. Preprint. [Version 1] doi: 10.64898/2026.06.24.733881 (PMC13321093; doi:10.64898/2026.06.24.733881)

Supplemental Figure 1

A

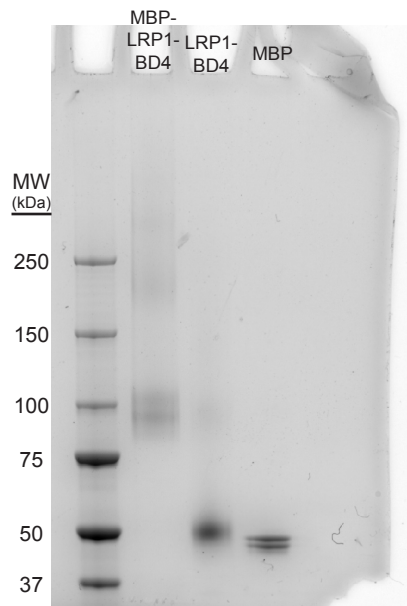

B

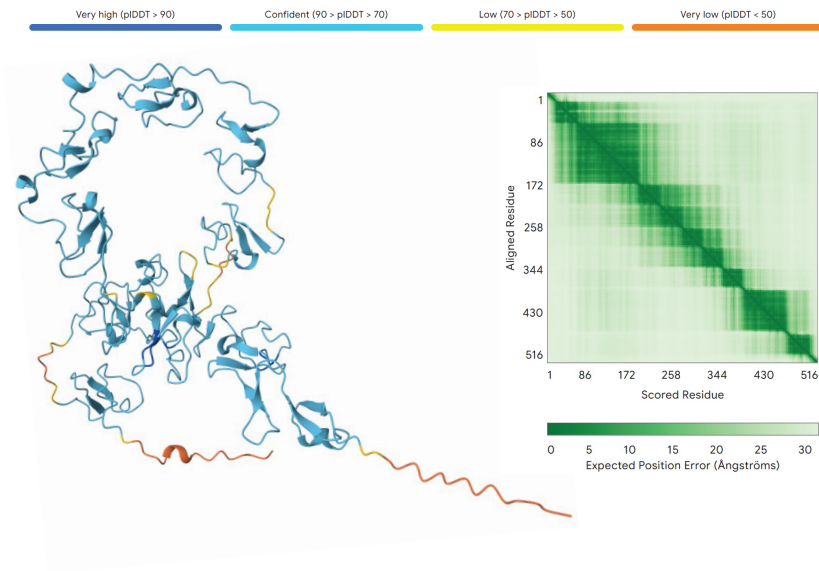

Supplement: Supplement 1 — Supplemental Figure 1. (A) SDS-PAGE Coomassie gel of MBP-LRP1-BD4 before (Lane 1) or after cleavage by thrombin (Lane 2 – LRP1-BD4; Lane 3 – MBP). (B) Alphafold3 prediction of LRP1-BD4. Model is colored by per-residue confidence score (pLDDT): dark blue (>90, very high), cyan (70-90, confident), yellow (50-70, low), and orange (<50, very low). Inset: Predicted Aligned Error (PAE) plot, where darker green indicates lower expected positional error (Å) between residue pairs. [file media-1.pdf]

Supplemental Figure 2

A

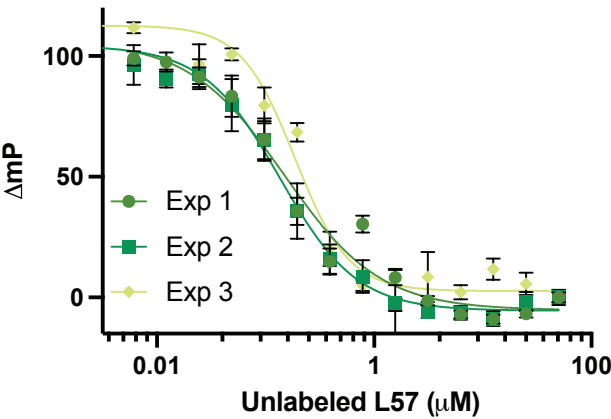

B

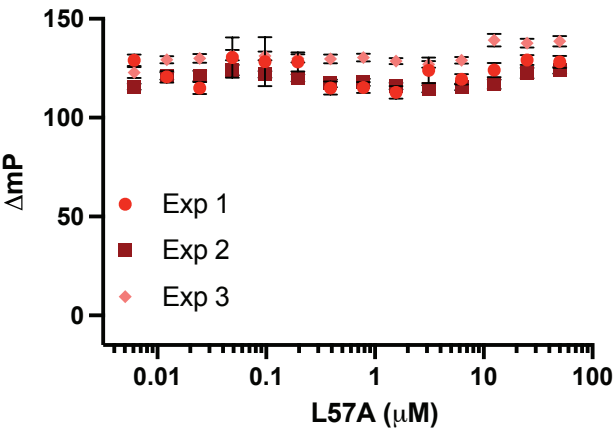

Supplement: Supplement 2 — Supplemental Figure 2. (A) Competition of unlabeled L57 with FITC-L57 for LRP1-BD4 binding. LRP1-BD4 (100nM) with L57-FITC (20nM), three independent experiments shown (mean±SD, technical triplicates) IC50 = 156nM (95% CI: 104-234nM, n=3 independent experiments). (B) Competition of unlabeled L57A with FITC-L57 (20nM) for LRP1-BD4 (100nM) binding, three independent experiments shown (mean±SD, technical triplicates). [file media-2.pdf]

Supplemental Figure 3

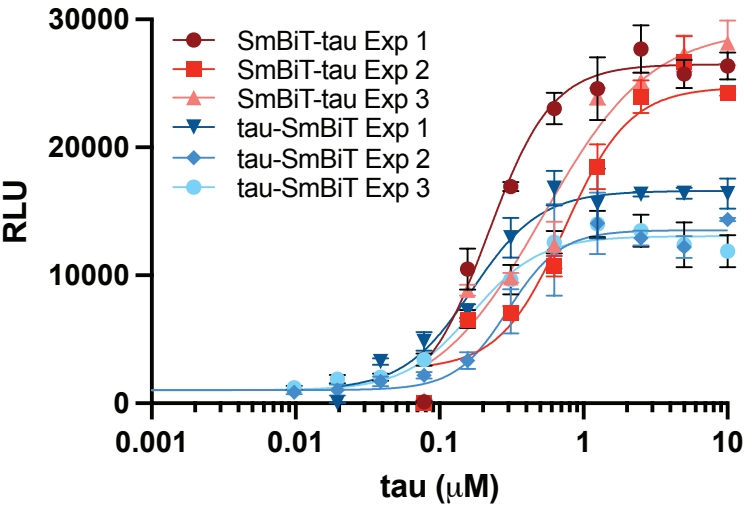

Supplement: Supplement 3 — Supplemental Figure 3. Titration of tau-SmBiT (C-term) or SmBiT-tau (N-term) with 10nM MBP-LgBiT-LRP1-BD4 produces luminescent signal (mean±SD, technical triplicates). Three representative experiments shown. [file media-3.pdf]

Supplemental Figure 4

A

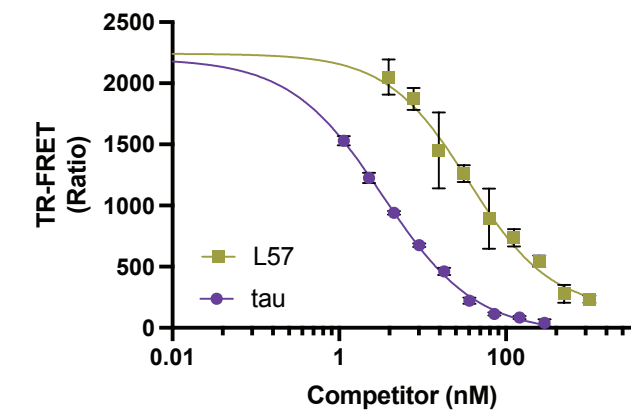

B

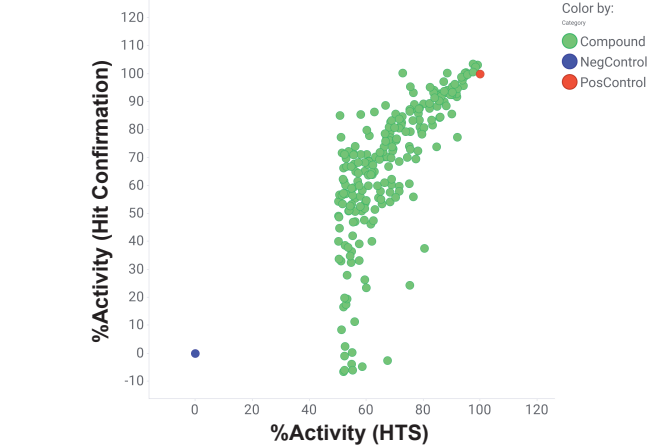

C

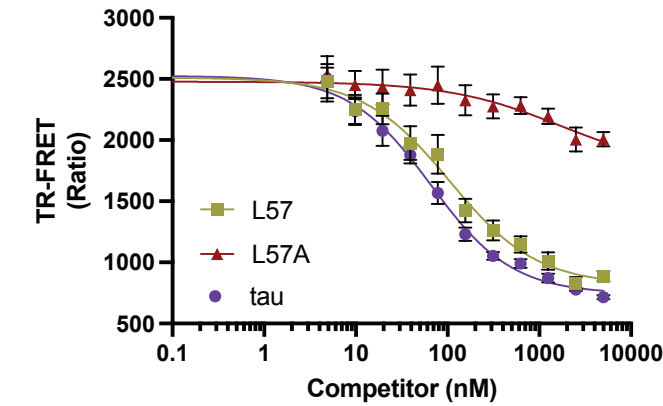

Supplement: Supplement 4 — Supplemental Figure 4. (A) LRP1-BD4-tau-647 TR-FRET competition with unlabeled tau and L57. Tau IC50 = 3.3nM (95% CI: 2.9[]ISP-CHK ALL]-3.9nM) and L57 IC50 = 36nM (95% CI: 23-66nM) (B) Hits from LRP1-BD4-tau TR-FRET screen show similar % Activity in the HTS and in the reconfirmation assay. (C) LRP1-BD4-L57 TR-FRET competition with unlabeled L57, tau, and L57A. L57 IC50 = 106nM (95% CI: 71-167nM), unlabeled tau IC50 = 66nM (95% CI: 50-87nM), and L57A was non-competitive. [file media-4.pdf]

Supplemental Figure 5

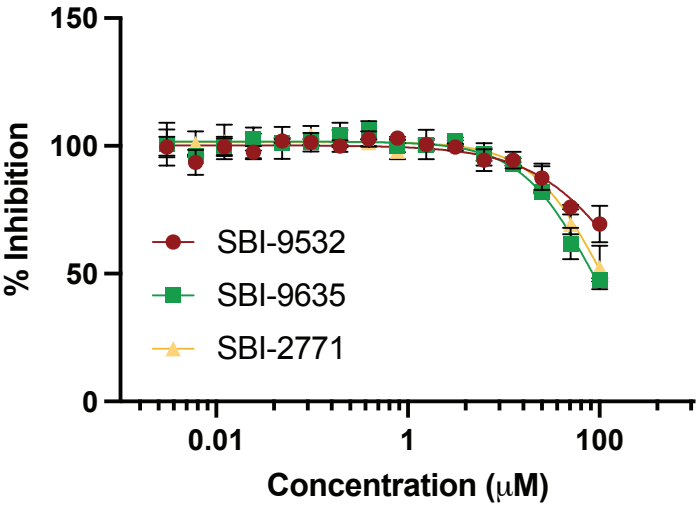

Supplement: Supplement 5 — Supplemental Figure 5. SBI-9532, SBI-9635, and SBI-2771 show minimal activity in the artifact assay. [file media-5.pdf]

Supplemental Figure 6

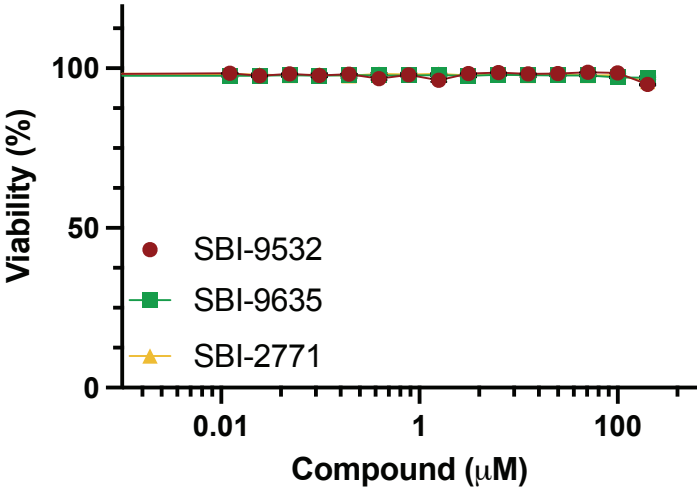

Supplement: Supplement 6 — Supplemental Figure 6. Viability of H4 neuroglioma cells with increasing concentrations of SBI-9532, SBI-9635, and SBI-2711. [file media-6.pdf]
